# Plantar acral melanoma: epidemiological, clinical, dermoscopic and histopathological features. A Brazilian cohort^☆^

**DOI:** 10.1016/j.abd.2024.03.006

**Published:** 2024-11-30

**Authors:** Lucas Campos Garcia, João Renato Vianna Gontijo, Flávia Vasques Bittencourt

**Affiliations:** a Department of Internal Medicine, Faculty of Medicine, Universidade Federal de Minas Gerais, Belo Horizonte, MG, Brazil; b Dermatology Unity, Hospital das Clínicas, Universidade Federal de Minas Gerais, Belo Horizonte, MG, Brazil

**Keywords:** Dermoscopy, Melanoma, Melanoma, amelanotic

## Abstract

**Background:**

Acral melanomas (AM) are rare and approximately two-thirds of them occur on the soles of the feet beeing more prevalent in black and Asian individuals. Data on this subtype of melanoma are scarce in the Brazilian population.

**Objectives:**

To describe and correlate the epidemiological, clinical, dermoscopic, and histopathological features of AM a.

**Methods:**

Single-center, retrospective and cross-sectional study, evaluating data from a 15-year period.

**Results:**

A total of 48 cases were included. Mean age was 62.54 years, with a predominance of women (62.5%). The percentage of amelanotic melanomas was higher among lighter skin patients (20% × 7.7%). Polychromia was the most prevalent finding (94.4%). The parallel ridge pattern (PRP) had a prevalence of 78% and a serrated pattern was associated with lower Breslow thickness (p = 0.041). Ulceration present on histopathological (p = 0.013) or dermoscopic (p = 0.047) evaluation was associated with greater Breslow thickness.

**Study limitations:**

Retrospective study with loss of data.

**Conclusion:**

Amelanotic tumors were more prevalent in ligther phototypes (20% × 7.7%). Polychromia was the most prevalent finding (94.4%) and ulceration observed on clinical or histopathological evaluation was associated with higher Breslow thickness (p = 0.013 and 0.047).

## Introduction

Acral lentiginous melanomas (ALM) were described by Reed et al. in 1976 as a distinct subtype of melanoma based on both location and histopathological criteria: preliminary phase of radial growth of atypical lentiginous cells, expansive growth in the papillary dermis or vertical growth in the reticular dermis, lymphocytic inflammatory infiltrate, signs of regression and aberrant cytology (elongated or epithelioid cells).^1^ Despite this definition, a major source of controversy was the fact that not all melanomas originating in the acral region showed these histopathological criteria. A percentage of them presented histologically as superficial spreading melanomas (SSM) or nodular melanomas (NM). In view of this, the term acral melanoma (AM) was proposed for all melanomas originating in the area of glabrous skin, where there are no hair follicles – palms of the hands, soles of the feet, and nail bed.^2^

AM is a rare entity, with an estimated incidence of 0.3/100,000 per year.^3^ It accounts for the majority of melanomas in populations without classic melanoma risk factors – low phototype, increased number of melanocytic nevi, history of sunburns and family history of melanoma – such as black and Asian individuals.^3^, ^4^ AM corresponds to approximately 50% of melanomas in the Asian population and it is estimated to be even more prevalent among blacks.^5^ In Caucasians, AM has a lower relative prevalence, 2% to 13%.^6^ In Brazil, the number of studies is scarce, and the largest published case series found a prevalence rate of 13.2% (n = 3,878) AM among the total melanoma cases .^7^ Despite marked differences when observing the relative incidence, AM has an unique characteristic among all other melanoma subtypes of presenting the same absolute incidence in all ethnicities.^8^ Plantar AM are the most frequent in relation to ungual and palmar melanomas and may account for more than two-thirds of the cases.^9^

AM pathogenesis remains controversial.^10^ Ultraviolet (UV) radiation does not seem to be implicated,^11^ and other risk factors, such as low phototype and increased number of melanocytic nevi, are not statistically related.^12^ No familial case of AM has been described to this date.^12^ Etiology may be multifactorial, with trauma remaining one of the possible risk factors.^8^ Recent studies have found a significantly higher incidence of AM in the areas where the soles support more weight, strengthening the hypothesis that trauma may be implicated in its pathogenesis.^13^, ^14^, ^15^, ^16^

Early diagnosis is the main key to effective treatment and better survival in all melanoma patients. The difficulty in finding consistent risk factors that would allow for more stringent monitoring of population subgroups, as is practiced for patients with multiple melanocytic nevi at risk for SSM, is an additional challenge in AM.

Dermoscopy has provided an invaluable contribution in pigmented lesions diagnosis, including acral lesions.^17^ Volar skin is characterized by dermatoglyphics (fingerprints), consisting of furrows and ridges. On dermoscopic examination, nevi tend to accumulate pigment along the furrows, determining a pattern called parallel furrow pattern (PFP), while melanomas accumulate pigment in the region corresponding to the ridges – parallel ridge pattern (PRP). 17, 18

Description of these aspects contributed to the early diagnosis of AM; however, its sensitivity and specificity remain controversial in the literature. In some case series, more than 80% of melanomas showed PRP,^18^, ^19^ with a sensitivity of 86%, specificity of 99%, and positive predictive value of 93% for AM diagnosis. However, Lallas et al. in two series with 118 and 131 AM patients reported the presence of PRP in 65.3% and 38.2%, respectively.^20^, ^21^ In the presence of PRP, it is necessary to consider surgical excision, despite of this pattern been identified in benign lesions, such as: Mongolian spots, acral pigmentation due to para-phenylenediamine and Peutz-Jeghers syndrome, pigmentation secondary to the use of chemotherapy drugs, subcorneal hematoma, plantar warts and congenital plantar nevi.^21^, ^22^, ^23^

**Figure 1:**
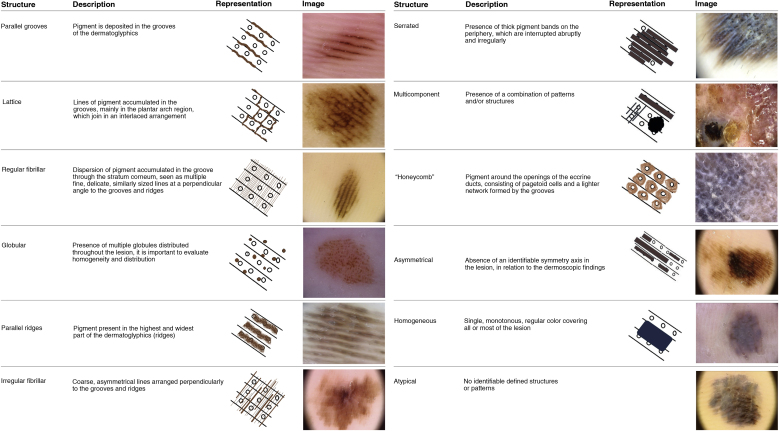
Dermoscopic patterns of acral pigmented lesions.

**Figure 2:**
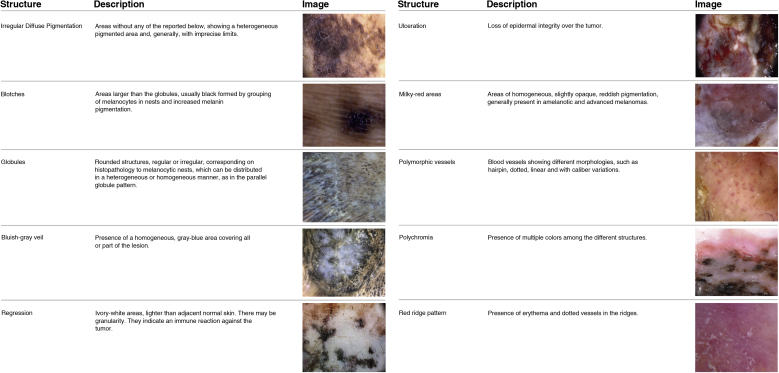
Dermoscopic structures of acral pigmented lesions.

Dermoscopic variants of PFP are associated with benign lesions, such as regular fibrillar, lattice-like, stepladder-like, double-line, pea-like patterns, in addition to the regular globular pattern. Other patterns described in the literature as associated with melanomas are asymmetric, red ridges, serrated, multi-component, honeycomb, irregular globular and irregular fibrillar patterns^18^, ^21^ (Fig. 1). Dermoscopic structures associated with AM have also been described, such as milky red areas, polymorphic vessels, irregular diffuse pigmentation, blotches, bluish-gray veil, regression, ulceration, and polychromia^18^, ^21^ (Fig. 2).

Histopathology is the gold standart for melanoma diagnosis. However histopathological diagnosis of pigmented lesions is challenging, since melanocytic nevi and early melanomas may share common characteristics, especially in acral sites.^22^ Classical histopathological criteria may not be sufficient to diagnose lesions that, clinically and dermoscopically, are suggestive of AM.^24^ Presence of melanocytic proliferation in the deep intermediate ridge, which anatomically corresponds to the ridge of the dermatoglyphs, is a histopathological criterion proposed to aid early diagnosis.^25^

AM is largely distinct genetically from other cutaneous melanomas. Genomically, a significant proportion – 38% to 55% – will be a wild type for the three mutations most often associated with melanomas: BRAF, NRAS, and NF1. One of the first differences identified in AM in relation to non-acral melanomas was the increased prevalence of KIT mutations: mutated in 10% to 40% of AM in some series and in less than 10% of non-acral melanomas.^8^, ^10^, ^24^

Treatment of choice for the primary site is surgery, however large studies that have determined surgical margins did not included AM in their cohorts .^26^ The high local recurrence rate described by some authors raises questions about the currently recommended margins.^26^

The challenges in diagnosing and treating AM, especially plantar melanomas, reflect in their worse prognosis when compared to other melanoma subtypes.^9^ Although AM is responsible for more than half of melanoma cases in black individuals, practically all available data is in predominantly Caucasian or Asian populations. It is important to emphasize that studies in populations such as Brazilian, with broad racial miscegenation, regarding the epidemiological, dermoscopic, clinical, and histopathological aspects of AM are still scarce and sparse. In addition, cultural factors, educational level and health education are other variables with very particular characteristics in the Brazilian population that can influence disease evolution.

## Methods

Data from patients with plantar AM were retrospectively collected. This is a cross-sectional, analytical observational study conducted at the Dermatology Clinic of Universidade Federal de Minas Gerais collected from 2005 onwards.

Inclusion criteria were patients with a histopathological diagnosis of plantar AM and clinical or dermoscopic photographic documentation over a 15-year period.

For each patient, information was retrieved regarding epidemiological features (age at diagnosis, sex, occupation, phenotypic characteristics, personal and family history of melanoma), clinical features (size, location, anatomical subunit, delimitation), dermoscopic (patterns, structures and colors) and histopathological characteristics (histopathological subtype, Breslow thickness, presence of ulceration, mitotic index). Each melanoma was marked on a plot representing the plantar surface, for subsequent analysis of what anatomical subunits were most affected.

The evaluation of clinical and dermoscopic images was performed by three different examiners. Photographs were taken using FotoFinder equipment or Canon Power Shot 3.2® or Nikon 1® digital cameras with a Dermlite DL3 and DL4 dermatoscope.

**Figure 3:**
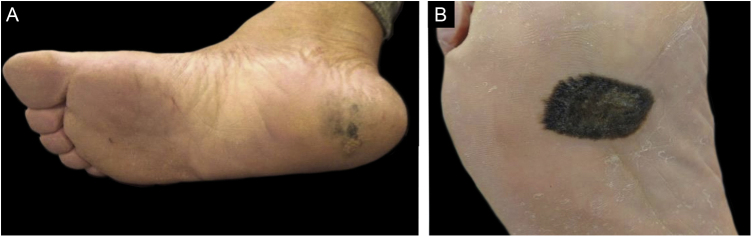
Variation in delimitation of acral melanoma. (A) Poorly delimited acral melanoma. (B) Well delimited acral melanoma.

As for the clinical aspects, tumors were classified according to their delimitation, precise and imprecise limits, and size. In lesions that occupied more than one anatomical subunit, the limits within the anatomical subunit where the largest area of ​​the tumor was located were considered (Fig. 3).

On histopathology, AM was divided into the subtypes ALM, SSM, and NM. *In situ* tumors were included in the study, and invasive lesions were categorized as thin (Breslow thickness < 1.0 mm) or thick (Breslow thickness ≥ 1.0 mm) melanomas. Data were obtained from histopathological reports available in their medical records.

Statiscal analyses of correlations between epidemiological, clinical, dermoscopic, and histopathological characteristics were performed. For the numerical variables the main descriptive statistics indexes were calculated (e.g., mean, median, standard deviation, coefficient of variation, and quartiles). The normality of each variable was tested in its original unit. For categorical variables, the relative and absolute frequencies were calculated.

The soles were divided into five anatomical subunits: calcaneus, arch, midfoot, forefoot, and edges. The edges and the plantar arch were considered areas that are less exposed to chronic trauma, while the others were considered areas exposed to chronic trauma. To assess the differences in the occurrence of melanoma of any type in the anatomical subunits and among weight bearing areas, the Chi-Square goodness of fit test was performed, assessing whether the proportions in each class were significantly different from a random distribution (without a pattern).

Association between two qualitative variables was verified using the Chi-Square test of independence, or Fisher's exact test for low expected frequencies (less than five). To assess the association between a qualitative variable and a quantitative one, the nonparametric Mann-Whitney test was applied. Mean prevalence of PRP was estimated from the ratio between the number of parallel ridge patterns observed divided by the total number of evaluated lesions. Confidence interval was constructed according to the methodology by Clopper and Pearson (1934). All statistical analyses were performed using R software version 3.6.1 (R Core Team, 2019). The study was approved by the Institutional Review Board and Ethics Committee of UFMG (# 4173035).

## Results

A total of 71 cases of AM diagnosed over a 15-year period were identified. Of these, 23 were excluded from statistical analysis due to insufficient data (incomplete medical records). Of the remaining 48 cases, 36 had dermoscopic images. The other 12 cases were admitted after the excision of the primary lesion and were included only in the analyses regarding epidemiological, clinical (location), and histopathological aspects.

Analysis of demographic data showed mean age of 62.54 years and median of 64 years, with a standard deviation (SD) of 16.5. The Shapiro-Wilk test did not allow for rejecting the normality of age distribution. There was a predominance of females, 62.5% (n = 30), in relation to male ; 37.5% (n = 18). Regarding phenotypic characteristics, the majority of the sample consisted of brown and black individuals 62.5% (n = 30). This information was not available in two patients and there were no patients of Asian descent. Only one of the participants had a previous history of melanoma, located in the vulva. Most of the tumors were greater than 2.0 cm in diameter (77.8%; n = 26). There was a slight predominance of tumors in the right plantar region (54.2%; n = 28), with no statistical significance (Table 1).

**Table 1:** Epidemiological, clinical and histopathological aspects of patients with acral melanoma.

**Mean age (years) (n = 48)**	62.54 ± 16.50	
**Gender (n = 48)**	Female	30 (62.5%)
	Male	18 (37.5%)
**Phototypes (n = 48)**	Unknown	2 (4.2%)
	Leukodermic	16 (33.3%)
	Brown/Melanodermic	30 (62.5%)
**Laterality (n = 48)**	Right	26 (54.2%)
	Left	22 (45.8%)
**Amelanotic (n = 36)**	No	32 (88.9%)
	Yes	4 (11.1%)
**Size (n = 36)**	<2.0 cm	8 (22.2%)
	>2.0 cm	28 (77.8%)
Limits*(n = 35)	Imprecise	23 (65.7%)
	Precise	12 (34.3%)
**Located at Weight** bearing **area (n = 48)**	Yes	27 (56.2%)
	No	21 (43.8%)
**Histological subtypes (n = 36)**	Acral lentiginous	31 (86.1%)
	Superficial spreanding	3 (8.3%)
	Nodular	2 (5.6%)
**Categorization by Breslow thickness (n = 36)**	*In situ*	19 (52.8%)
	Thin	2 (5.6%)
	Thick	15 (41.7%)

* One of the tumors could not have its delimitation classified (n = 35).

**Figure 4:**
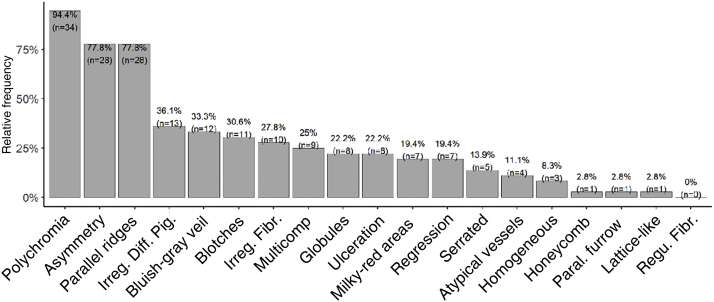
Prevalence of dermoscopic findings.

Dermoscopic analysis revealed polychromia as the most prevalent finding (94.4%). PRP had a prevalence of 78% with a 95% confidence interval between 61% and 90%. Asymmetry was as prevalent as PRP. Patterns classically associated with benign lesions, such as PFP (n = 1; 2.8%), lattice-like pattern (n = 1; 2.8%), and fibrillar pattern (n = 0), were the least prevalent. The serrated pattern was found in five tumors (13.9% of the sample). Other patterns and structures classically related to melanoma had intermediate prevalence, such as irregular diffuse pigmentation (IDP) in 36.1% (n = 13), bluish-gray veil in 33.3% (n = 12), irregular fibrillar pattern in 27.8% (n = 10), multicomponent in 25% (n = 9) and ulceration in 22.2% (n = 8) of the cases (Fig. 4).

The most prevalent histopathological subtype was ALM in 86.1% (n = 31), followed by SSM in 8.3% (n = 3) and NM in 5.6% (n = 2). The majority (52.8%; n = 19) were *in situ* lesions, followed by thick lesions in 41.7% (n = 15) and thin lesions in 5.6% of the cases (n = 2; Table 1). Mean Breslow thickness of invasive lesions was 3.84 ± 3.11 and the median was 2.9. Histopathological ulceration was detected in 12.1% of the cases. Mitoses were observed in 24.2% of the tumors, while the mean mitotic index in positive cases was 8.3, and in general it was 1.63.

**Figure 5:**
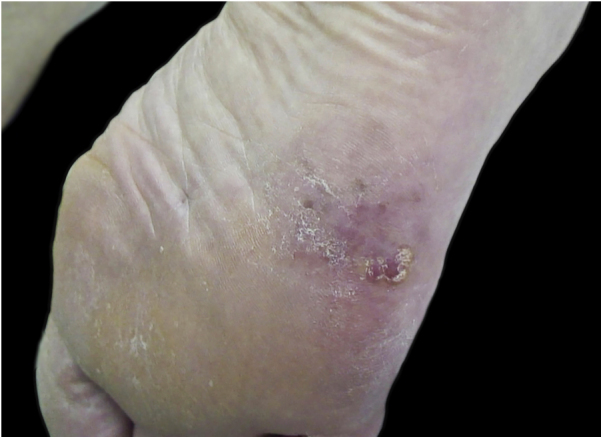
Hypomelanotic plantar acral melanoma.

Although it did not reach statistical significance (p = 0.305), the percentage of amelanotic melanomas (Fig. 5) was higher among white patients compared to those with higher phototypes (20% vs. 7.7%). No significant differences were observed in the frequency of tumor occurrence in weight bearing areas (56.2%; n = 27) or non-weight bearing areas (43.8%; n = 21; Table 1). Regarding anatomical location, there was a marginally significant difference (p = 0.076), with a tendency towards greater concentration in the calcaneus.

**Figure 6:**
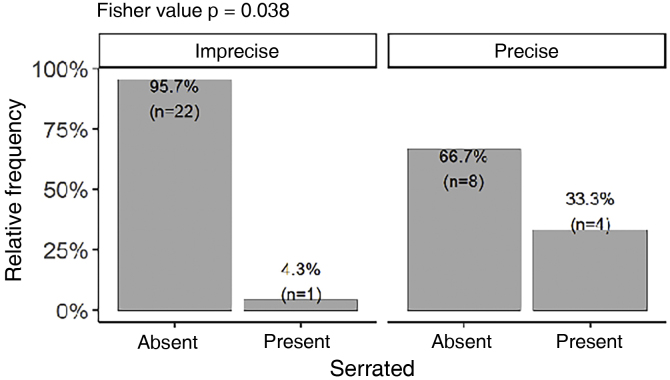
Prevalence of serrated pattern in tumors with precise and imprecise limits (n = 35).

The serrated pattern was associated with lower Breslow thickness (p = 0.041) and was present more frequently in tumors with precise limits (Fig. 6). Presence of ulceration, whether detected on histopathological (p = 0.013) or dermoscopic (p = 0.047) , was associated with higher Breslow thickness. There was no statistical difference between Breslow thickness and skin color (p = 0.82) or gender (p = 0.247).

**Figure 7:**
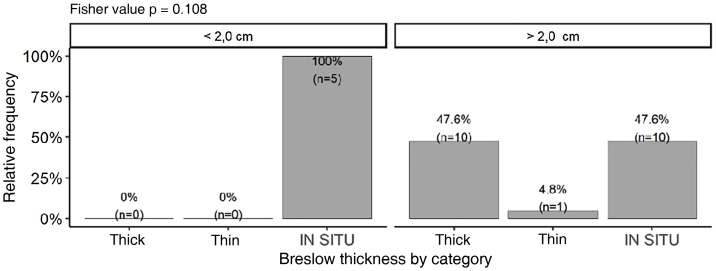
Breslow thickness staging according to tumor size (n = 26).

Although no statistically significant difference was observed, tumors smaller than 2.0 cm (n = 5) had a tendency to present lower Breslow thickness ​​(p = 0.108). On the other hand, a significant percentage of tumors larger than 2.0 cm (47.6%; n = 10) were still diagnosed as *in situ* tumors (Fig. 7). Majority of tumors larger than 2.0 cm (85.7%) showed PRP, while only 50% of tumors smaller than 2.0 cm exhibited this finding. There was no association between the presence of PRP and staging by Breslow thickness (p = 0.678).

## Discussion

The epidemiological data presented here is similar to those described by several authors. Participants mean age was 62.54 years (SD ± 16.5),similar to the mean of 62.9 years found in the global literature. This is also similarwhen compared specifically with the mean age of Brazilian studies,that was 63.6 years The slight predominance of female patients has already been described in the literature, as of 54.8% females.^7^, ^11^, ^20^ This predominance was also observed in our study, 62.5% (n = 30), in relation to male patients 37.5% (n = 18). The difference was not significant, although there have been reports of significant discrepancies between genders.^27^

Most participants 62.5% (n = 30) had higher phototypes. No patients of Asian descent were identified, this ethnicity in the study region is sparse and may be the reason for this result. One of the participants had a previous history of melanoma in another site (vulvar region), which is an extremely rare association. As it is classically documented for AM, most participants did not have a family history of melanoma; however, the daughter of one of the participants had an AM on the thumbnail.

Clinically most tumors (77.8% (n = 28) were larger than 2.0 cm in diameter. This data reinforces the challenge of providing an early diagnosis in AM, and consequently a better prognosis. Although dermoscopy is a very useful tool for the differential diagnosis of acral pigmented lesions, patients with lesions larger than 2.0 cm partially limit its usefulness, as these are unlikely to have a diagnosis other than AM. Other groups of authors have already identified that the introduction of dermoscopy did not directly impact the survival of these patients,^28^ probably for the same reason: predominance of clinically advanced cases.

Chronic trauma role in AM pathogenesis remains controversial. In our study, AM was more prevalent in weight bearing areas, which are more exposed to chronic trauma, than in the others. However, the difference (weight bearing areas: 56.2% n = 27 vs. non-weight bearing areas 43.2% n = 21) was not significant, similar to the findings reported by Costello et al.^29^ Another recent series, however, found an association between areas of chronic mechanical stress and the incidence of AM.^30^ When evaluating tumor frequency at anatomical subunits, there was a marginally significant difference (p = 0.076), with a tendency towards a higher concentration in the calcaneus.

In the present study, amelanotic tumors accounted for 11% of the total, lower than in the literature, which varies from 24% to approximately one-third of cases.^31^ The percentage of amelanotic melanomas was higher in lower phototypes (20%; n = 2), probably due to sample size and the difference was not statistically significant (p = 0.305). The hypothesis that patients with lower phototypes are more likely to have acral amelanotic or hypomelanotic tumors should be investigated in future studies since this relationship is well-established in the literature for other melanoma subtypes.^32^

PRP had a prevalence of 77.8% (95% CI 61% and 90%), which is similar to what was found in a South Korea case series published by Mun et al. (85.3%)^19^ and from Japan by Saida et al. (86%),^18^ but is higher than that described by Lallas et al. (38.2%).^21^ Although most studies indicate this finding as the most specific for the diagnosis of AM, a prevalence rate as low as that described by this last group could impact the PRP negative predictive value. There was no association between the presence of PRP and staging by Breslow thickness (p = 0.678). However PRP was observed more frequently in lesions larger than 2.0 cm. This data is somewhat conflicting with those found by Saida et al, who associated PRP with earlier lesions,^22^ despite of other studies that have shown a higher prevalence of PRP in larger lesions.^33^ In our study, larger tumors had a higher chance of presenting PRP, even if only focally.

Polychromia was the most prevalent dermoscopic finding (94.4%; n = 34), followed by asymmetry and PRP (both present in 77.8%; n = 28 of the lesions). It is worth mentioning that polychromia and asymmetry on clinical evaluation are classic and established criteria for the diagnosis of melanoma. Polychromia has been previously identified as a highly prevalent finding, both in AM,^34^ and other melanoma subtypes, such as SSM (81%)^35^ and ungual AM (72.7%).^36^ Another series had previously reported a high prevalence (88%) of asymmetric lesions.^37^ IDP was found in 36.1% (n = 13) of tumors and was not associated with higher Breslow thickness. This differs from those described by Saida, et al., who found a prevalence of 90% and an association of the findings with more advanced tumors.^18^ However, other series such as the ones published by Braun et al. and Ozdemir et al. had already reported lower prevalence rates of IDP, 20.5% and 28.3%, respectively.^4^, ^36^

Interestingly, the serrated pattern was associated with lower Breslow thickness. We have not identified any other description of this association to date and further studies need to be carried out to confirm it. This serrated pattern was also significantly associated with well-defined lesions, perhaps because it occurs at the periphery of the lesion, where the limits must be well-defined for this pattern to be identified. Other analyses regarding the distribution of dermoscopic findings in relation to tumor delimitation and size, Breslow thickness, anatomical subunit, and occurrence in weight bearing and non-weight bearing areas were not significant. It is worth noting that some authors have published associations between dermoscopy and histopathology or clinical findings, such as those by Saida et al, Braun et al. and Lallas et al.^4^, ^20^, ^21^, ^22^, ^24^, ^38^

Presence of ulceration, identified by histopathology (p = 0.013) or dermoscopy (p = 0.047), was associated with higher Breslow thickness. This correlation is widely supported by previously articles in which the presence of ulceration appears as a predictor of advanced lesions and, consequently, of poor prognosis.^11^ The percentage of ulcerated tumors, 22% (n = 8), was lower than the average of the main studies available in the literature (51.73%), among which the lowest reported value was 33.3%.^28^

The percentage of tumors classified as ALM, 86.1% (n = 31), was higher than the average of main studies in the literature (64.46%) and the average of previously published national studies (36.03%). It is worth mentioning that some of these studies^3^, ^39^ included melanomas of the dorsal portions of the hands and feet, which could contribute to this discrepancy. Another group found a prevalence of ALM (85.9%) close to that found in the present sample.^40^

Regarding tumor thickness, there was a tendency to dichotomize between *in situ* lesions (52.8% (n = 19) and thick ones (41.7%; n = 15). Mean Breslow thickness of invasive lesions was 3.84 mm(±3.11), very close to the average of main studies (4.08 mm) and slightly lower than the average of previously published brazilian studies (4.57 mm). Since Breslow thickness is the most important isolated prognostic predictor, this data allows the assumption that there was no significant diagnostic delay in relation to other research centers. However, the high number of *in situ* lesions (52.8%; n = 19) even with a high prevalence of tumors larger than 2.0 cm (77.8%; n = 28) may indicate that AM has a relatively long radial growth phase, similar to what occurs with lentigo maligna melanoma and that worse prognosis is primarily associated with diagnostic delay. This delay is aggravated by the fact that this lesion is often asymptomatic, its practically unknown to the general population and also by health professionals. Some groups have reported that when adjusting for the stage/thickness of the tumors, there is no difference in survival; therefore, AM would not have a more aggressive biological behavior, only suffer a longer diagnostic delay.^6^

Identification of risk factors, information dissemination, and education about AM are important for its diagnosis at earlier clinical stages. So it is necessary to prioritize this information in prevention campaigns aimed at the population and reinforce physicians education, residents, and students regarding the importance of performing a total physical examinations of the soles . Dermoscopic training should be emphasized to assist in the differentiation from melanocytic nevi, which are widely prevalent.

The present study is a single-center study and has several limitations, mainly due to its retrospective design. Among them, one can highlight the missing data and the quality of some photographic records. This was a small, convenience sample, and may not represent a population with broad miscegenation such as the Brazilian population. For these reasons, further studies are needed to corroborate the presented data.

## Conclusions

The present sample of plantar AM reveals epidemiological data compatible with previously described in the literature, such as mean age of involvement in the sixth decade of life and a slight predominance of females. Patients with higher phototypes, i.e., brown and black individuals, were more affected, a particularity expected in the Brazilian population.

Clinically, the tumors predominated in areas of chronic trauma, however this difference was not significant. There was a marginally significant tendency for lesions to be concentrated in the calcaneus. There was a predominance of large tumors, with more than 2.0 cm in diameter.

Prevalence of PRP was 77.8% (95% CI 61% and 90%), approaching the values ​​described in Asian series. However, polychromia was the most prevalent dermoscopic finding. Other analyses of dermoscopic findings did not reach statistical differences, such as prevalence in subunits, weight bearing areas, size and delimitation. Presence of the serrated pattern was associated with good delimitation.

There was a dichotomization between *in situ* and thick tumors. The most frequent histopathological subtype was ALM, with a frequency above the average of previous studies. There was no significant diagnostic delay in relation to the mean Breslow thickness, which was comparable to that of other studies. Histopathological and dermoscopic ulceration was associated with higher Breslow thickness. On the other hand, the serrated pattern was associated with lower Breslow thickness. The present study contributes new information on plantar AM in the Brazilian population and supplements the scarce literature, not only in Brazil but also in Latin America.

## Financial support

None.

## Authors' contributions

Lucas Campos Garcia: Drafting and editing of the manuscript, critical review of important intellectual content and approval of the final version of the manuscript.

João Renato Vianna Gontijo: Drafting and editing of the manuscript, critical review of important intellectual content and approval of the final version of the manuscript.

Flávia Vasques Bittencourt: Drafting and editing of the manuscript, critical review of important intellectual content and approval of the final version of the manuscript.

## Conflicts of interest

None declared.
